# Age-dependent gene expression trajectories during early childhood in children at increased risk for type 1 diabetes

**DOI:** 10.1038/s41435-025-00324-8

**Published:** 2025-03-20

**Authors:** Ivo Zeller, Andreas Weiss, Sandra Hummel, Anette-Gabriele Ziegler, Ezio Bonifacio

**Affiliations:** 1 Institute of Diabetes Research, Helmholtz Munich, German Center for Environmental Health, Munich, Germany; 2 Forschergruppe Diabetes e.V. at Helmholtz Munich, German Research Center for Environmental Health, Munich, Germany; 3 Forschergruppe Diabetes, School of Medicine, Klinikum rechts der Isar, Technical University Munich, Munich, Germany; 4 Technische Universität Dresden, Center for Regenerative Therapies Dresden, Dresden, Germany; 5 Paul Langerhans Institute Dresden of the Helmholtz Munich at University Hospital Carl Gustav Carus and Faculty of Medicine, TU, Dresden, Germany

**Keywords:** Gene expression, Autoimmunity

## Abstract

Early childhood is a period of rapid growth and immune system development. It is also critical for type 1 diabetes (T1D) autoimmunity, which has a peak incidence between 1 and 2 years of age. Here, we investigated age-related longitudinal gene expression changes in peripheral blood mononuclear cells from children aged 3 months to 3 years who had an increased genetic risk for T1D, aiming to delineate gene expression trajectories and identify patterns potentially linked to the development of islet autoimmunity. We found 2 432 genes (12.5% of analyzed genes) to exhibit significant temporal dynamics in the first 3 years of life. These genes were grouped into six major clusters each demonstrating distinct expression trajectories of consistent increase or decrease with age, as well as U-shaped, and inverted U-shaped age-related patterns. Notably, genes in clusters with U-shaped expression trajectories, which mirrored the incidence of islet autoantibodies, were enriched for T1D susceptibility genes, particularly within the Major Histocompatibility Complex (MHC) region. This study underscores the dynamic nature of gene expression in early childhood and its potential connection to T1D risk.

## Introduction

Early childhood represents a phase marked by substantial growth and exposure to new challenges for the immune system [1–3]. It is also a period of heightened susceptibility to immune-mediated diseases, such as allergy and autoimmunity [4]. The initial years of life pose a significant risk for autoimmunity that leads to type 1 diabetes (T1D). Children who are at increased genetic risk for type 1 diabetes show a peak incidence of islet autoantibodies at around one year of age, followed by an exponential decline in the risk of autoimmunity thereafter [5, 6]. Additionally, children manifesting islet autoimmunity at an early age progress more rapidly to clinical diabetes compared to those developing autoimmunity later in childhood [5, 7]. Consequently, comprehending immune cell trajectories during the first years of life holds potential significance in identifying risk factors for immune-mediated diseases. Recent studies have reported gene expression in relation to islet autoantibody seroconversion or progression to T1D, but most of these have not assessed agerelated changes [8, 9]. The objective of this study was to investigate whether there are age-related changes in immune cells that mimic or mirror the risk of islet autoimmunity. To address this, we analysed longitudinal peripheral blood transcriptomic data collected from age 3 months to 3 years in children at increased genetic risk for type 1 diabetes to delineate age-related gene expression trajectories.

## Methods

### Cohort

The study was performed on 395 samples from 108 children who had a genetic predisposition to type 1 diabetes (T1D) and were participants in the BABYDIET study [7], which followed children who had at least one first-degree relative with T1D and a T1D susceptible HLA DR-DQ genotype from approximately 3 months of age with collection of venous blood samples every 3 months until they reached 3 years of age (Supplemental Fig. 1a, Supplemental Table 1). The age distribution of samples was similar across HLA genotypes (Supplemental Fig. 1b). Of the 108 children, 26 developed persistent islet autoantibodies (18 multiple islet autoantibodies) and 21 have developed T1D. The BABYDIET study was approved by the ethics committee of Ludwig-Maximilians University in Munich, Germany (Ethikkommission der Medizinischen Fakultät der Ludwig-Maximilians-Universität, No. 329/00). Parents or legal guardians provided signed informed consent for their children to participate in the BABYDIET study.

### Peripheral blood mononuclear cell microarray gene expression data

Microarray gene expression data used for the current analyses were previously generated from peripheral blood mononuclear cells (PBMC) and reported [10]. These data were generated using Titan Affymetrix Human Gene 1.1 ST arrays, which comprise over 750,000 unique 25-mer oligonucleotide probes.

### Differential gene expression analysis and trajectory clustering

Data were summarized by gene-level probe sets and normalized using quantile normalization.

Differential expression analysis was conducted using the limma package in R [11]. A linear mixed effects model with cubic splines and 3 degrees of freedom was applied to account for the temporal nature of the data. This model incorporated time, sex, and season as fixed effects, while accounting for the random effect of subject-specific intercepts. The cubic spline term was used to capture potential non-linear changes in gene expression over time. The model discerned genes with temporal expression dynamics, designating a gene as differentially expressed when both the minimum absolute fold change over time exceeded 10% and the adjusted *p*-values were below 0.05, following correction using the Benjamini-Hochberg method. Gene expression trajectories were clustered using hierarchical clustering with Kendall’s tau correlation as the similarity measure, followed by manual assignment into larger clusters (see Supplemental Methods).

Gene set enrichment analysis was performed with the R package clusterProfiler [12]. A multiple *p*-value correction was not applied due to the exploratory nature of the study. Terms with p-values below 0.05 were considered statistically significant, and the resulting lists were summarized as treemaps using REVIGO [13]. The box size in the treemaps reflects the enrichment score for the term. The word size in the gene word clouds reflects the number of significantly enriched GO terms associated with each gene, indicating its importance. The larger the word, the more diverse functions are linked to that gene. To identify T1D susceptibility genes, we queried the Harmonizome database, which includes 144 distinct genes associated with T1D, of which 119 were in the array.

Calculations for enrichment were performed using a two-sided Fisher’s exact test.

To identify the factors contributing to gene expression variation, we utilized the variancePartition package [14].

Interaction terms incorporating age in regression analyses were utilized to discern differences in gene expression patterns across sex and HLA genotypes.

## Results

### Gene expression trajectories

**Fig. 1:**
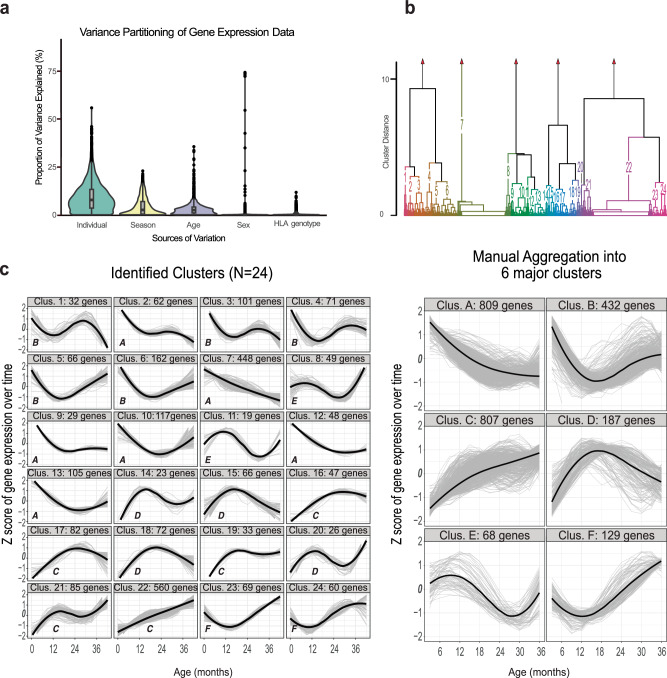
Factors influencing gene expression and the hierarchical clustering and aggregation of gene expression trajectories. **a** A violin plot depicting the proportion of variance in gene expression explained by individual, season, age, sex, and HLA genotype, across all genes. The partitioning highlights the relative contribution of each variable to gene expression variability. **b** Dendrogram depicting hierarchical clustering of gene expression trajectories over time, identifying 24 distinct clusters based on trajectory correlations. **c** (Left) Identified Clusters: Gene expression patterns over time for the 24 clusters, with each plot showing the Z-score of gene expression over age (in months) and the general trend within a cluster. The number of genes in each cluster is indicated. (Right) Manual Aggregation: The 24 identified clusters were manually aggregated into 6 major clusters (A-F) based on similarities in expression patterns. Each aggregated cluster shows the Z-score of gene expression over time, with the number of genes in each major cluster noted.

Gene expression was mainly influenced by interindividual differences, age, and season and less so by sex and HLA genotype (Fig. 1a). After adjusting for sex and season, a total of 2 432 (12.5%) genes out of 19,424 genes included in the microarray analysis exhibited temporal gene expression dynamics in peripheral blood mononuclear cells (PBMC) over the first 3 years of life (Fig. 1b, c, Supplemental Table 2, Supplemental Fig. 2). No significant interactions were observed between sex and age trajectories and between HLA DR genotype and age trajectories for the 2 432 genes. Hierarchical clustering was used to categorize the trajectory patterns into 24 clusters (Fig. 1c), which were aggregated manually into 6 major clusters according to their shape into ‘decreasing with early age’ (cluster A), ‘U-shaped’ (B), ‘increasing with early age’ (cluster C), ‘inverted U-shape’ (cluster D), ‘stable’ (cluster E) and ‘stable or declining followed by an increase’ (cluster F).

### Gene ontology enrichment analysis of trajectory clusters

**Fig. 2:**
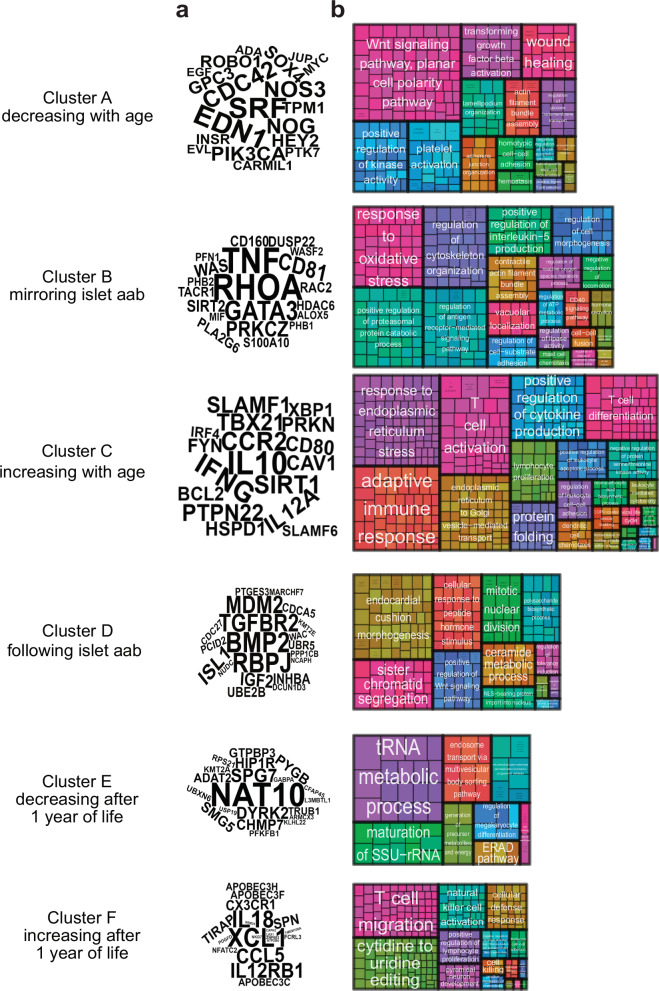
Functional analysis of trajectory clusters with various trends. **a** Word Clouds for each cluster: Key genes associated with the pathways of each cluster. The importance of each gene is indicated by word size, which is proportional to the number of occurrences within enriched Gene Ontology Biological Process (GO BP) terms. **b** Treemaps of enriched GO BP terms for each cluster, showing the functional enrichment of biological processes.

Gene ontology enrichment analysis was performed for clusters A to F (Fig. 2). Multiple immune-related pathways were identified in clusters C and F, which showed increased expression with age. Genes with the strongest importance in Cluster C included those encoding for cytokines *IFNG*, *IL10*, and *IL12*, the Th1 cell transcription factor *TBX21* and the type 1 diabetes susceptibility genes *PTPN22* and *CTLA4*. The genes indicated among the most important in cluster F included chemokine superfamily genes *XCL1* and *CCL5* as well as the cytokine *IL18* and the cytokine receptor *IL12RB*.

Pathways annotated from genes declining from early age in cluster A included developmental pathways involving WNT signaling and TGFB activation as well as genes such as *CDC42* that are important for cytoskeleton and therefore phagocytosis and cell adhesion. Genes in cluster E were annotated to RNA processing and stability, and cellular energy and sorting.

Pathways annotated to genes with U-shaped expression (cluster B) and inverted U shape expression (cluster D), akin to the early trajectory of blood glucose [15] and the islet autoantibody incidence curve, respectively were of particular interest. Cluster B genes were enriched for multiple pathways involved in oxidative stress and metabolism as well as intracellular processes. Also prominent was the regulation of antigen receptor-mediated signaling pathway, which is relevant to T and B cell activation. Notable genes included *RHOA* that is involved in cytoskeletal dynamics, cell cycle, and cell migration [16], and the multifunctional proinflammatory cytokine *TNF*, which is also a type 1 diabetes susceptibility gene [17]. The subgroup cluster 6 within Cluster B was enriched for genes associated with glucose metabolism, insulin secretion and pancreatic islet cell differentiation (Supplemental Fig. 3, Supplemental Table 3). Enriched terms for the Cluster D genes included peptide hormone response pathways and cell division pathways, among others. Genes include *BMP2* and *TGFR2*, both involved in the differentiation of regulatory T cells [18].

### T1D susceptibility genes

In total, 119 T1D susceptibility genes were included in the probe sets. Of these, 26 (21.8%) were among the genes with temporal gene expression dynamics, representing a significant enrichment (OR, 2.0; 95% CI, 1.3-3.2; *p* = 0.002). Enrichment was observed for the U-shaped clusters B, which included 9 T1D susceptibility genes (OR, 3.8; 95% CI, 1.7–7.5; *p* = 0.001; Supplemental Fig. 4) and F, which included 3 T1D susceptibility genes (OR, 4.0; 95% CI, 0.8–12.4; *p* = 0.04).

Of the T1D susceptibility genes identified in cluster B, 6 were within the MHC region of chromosome 6. We, therefore, looked for enrichment of the 159 genes located in the MHC region spanning chr6: 28,510,120 to 33,480,577 (Hg38). Significant age-related dynamics were observed for 31 (19.5%) of these genes (OR, 1.7; 95% CI, 1.1–2.5; *p* = 0.01). Enrichment was observed for cluster B, which included 13 genes within the MHC region (OR, 4.0; 95% CI, 2.1–7.1; *p* < 0.0001), but not other clusters (Supplemental Table 4; Supplemental Fig. 4).

## Discussion

Early life is a period of dynamic growth and adaptation to the environment. Here, we find that 12.5% of genes show early life dynamic expression changes in PBMC. Changes include increased expression of genes with age, decreased expression with age and both U- and inverted U-shaped expression resembling trajectories of blood glucose and of type 1 diabetes-associated autoimmunity incidence curves, respectively.

Age-related increases (807 genes) and decreases (809 genes) over the first two to three years of life were the most common gene expression trajectories. Expected changes include those in genes related to the maturation of the immune system that acquires memory through antigen exposure. A particularly dynamic genomic region with respect to gene expression was the MHC [19], which comprises 159 genes of which 31 showed age-related expression changes. Many of these genes are involved in immune responses. Of potential relevance to the age 1-year peak incidence of islet autoimmunity, there was a two-fold enrichment of T1D susceptibility genes, including MHC genes, among those with age-related expression changes and this enrichment was pronounced for genes that had trajectories mirroring the islet autoantibody incidence (Cluster B).

The study had several limitations. First, the cohort included children from Germany with a high genetic risk for type 1 diabetes based on family history of type 1 diabetes and HLA DR-DQ genotype. It is not possible, therefore, to determine whether the age-related trajectories are generalizable to children without an increased T1D genetic risk or are features of T1D genetic susceptibility. Similarly, the gene trajectories and associated pathways are likely to be affected by and reflect the many exposures and changes that occur in early childhood, some of which will act via epigenetic mechanisms, and may have little or no relation to the early susceptibility for islet autoimmunity or changes in metabolism that are observed in this age period [15, 20]. Second, the starting material was PBMC and we cannot attribute changes to particular cell types. Third, data were microarray-based and findings may differ to RNAseq data [9].

In summary, we observed age-related gene expression changes in PBMC in a large number of genes during the first years of life in children with an increased risk for T1D. Further studies are required to determine whether these changes are related to specific cell types involved in the pathogenesis of T1D and if these changes increase the risk of islet autoimmunity.

## Supplementary information

Supplementary information

Supplemental Table 2

Supplemental Table 3

## Data Availability

All the gene expression data have been documented in ArrayExpress (http://www.ebi.ac.uk/arrayexpress) under the accession number E-MTAB-1724.
