# Supplementary material for: Age-dependent gene expression trajectories during early childhood in children at increased risk for type 1 diabetes
Source: Genes Immun. 2025 Mar 20;26(2):173–7. doi: 10.1038/s41435-025-00324-8 (PMC12006016; doi:10.1038/s41435-025-00324-8)
Supplement: Supplementary file 1 — Supplementary information [file 41435_2025_324_MOESM1_ESM.docx]

**Age-Dependent Gene Expression Trajectories During Early Childhood in Children at Increased Risk for Type 1 Diabetes**

Ivo Zeller^1^, Andreas Weiss^1^, Sandra Hummel^1^, Anette-Gabriele Ziegler^1,2,3^, Ezio Bonifacio^4,5^

**Supplementary information**

**Supplementary Methods**

**Manual assignment of the 24 clusters to major cluster groups.**

A nadir was defined as a drop of at least 30% between the minimum and maximum expression, followed by a subsequent rise of at least 30% of the difference between the starting point and the nadir, occurring before age 3 years. Conversely, a peak was defined as a rise of at least 30% between the minimum and maximum expression, followed by a subsequent drop of at least 30% of the difference from the peak to the starting point, also before age 3 years.

Clusters were assigned to the U-shaped major cluster B if they met the following criteria: (1) the highest expression was observed at the youngest age, (2) the mean expression curve showed a nadir between 12 and 24 months of age, and (3) there was a subsequent rise in expression of at least 30% of the maximum difference between the start and the nadir. Clusters with U-shaped trajectories where the highest expression was not at the youngest age were categorized as major cluster F.

Clusters were assigned to the inverted U-shaped major cluster D if: (1) the lowest expression was observed at the youngest age, (2) the mean curve displayed a peak between 12 and 24 months, and (3) there was a subsequent decline of at least 30% of the maximum difference between the start and the peak. Clusters with inverted U-shaped trajectories where the lowest expression was not at the youngest age were classified into major cluster E.

**Supplementary Figure 1: Overview of the BABYDIET Cohort and Sample Collection Timeline**. (a) Age distribution of samples for 108 children in the BABYDIET cohort. (b) HLA genotype distribution in samples by age groups: The bar graph illustrates the percentage distribution of samples from children by HLA genotypes (DR 3/4, DR 4/4 - DQ8, DR 3/3, and other) across four age intervals (0-9, 10-19, 20-29, and 30-39 months).
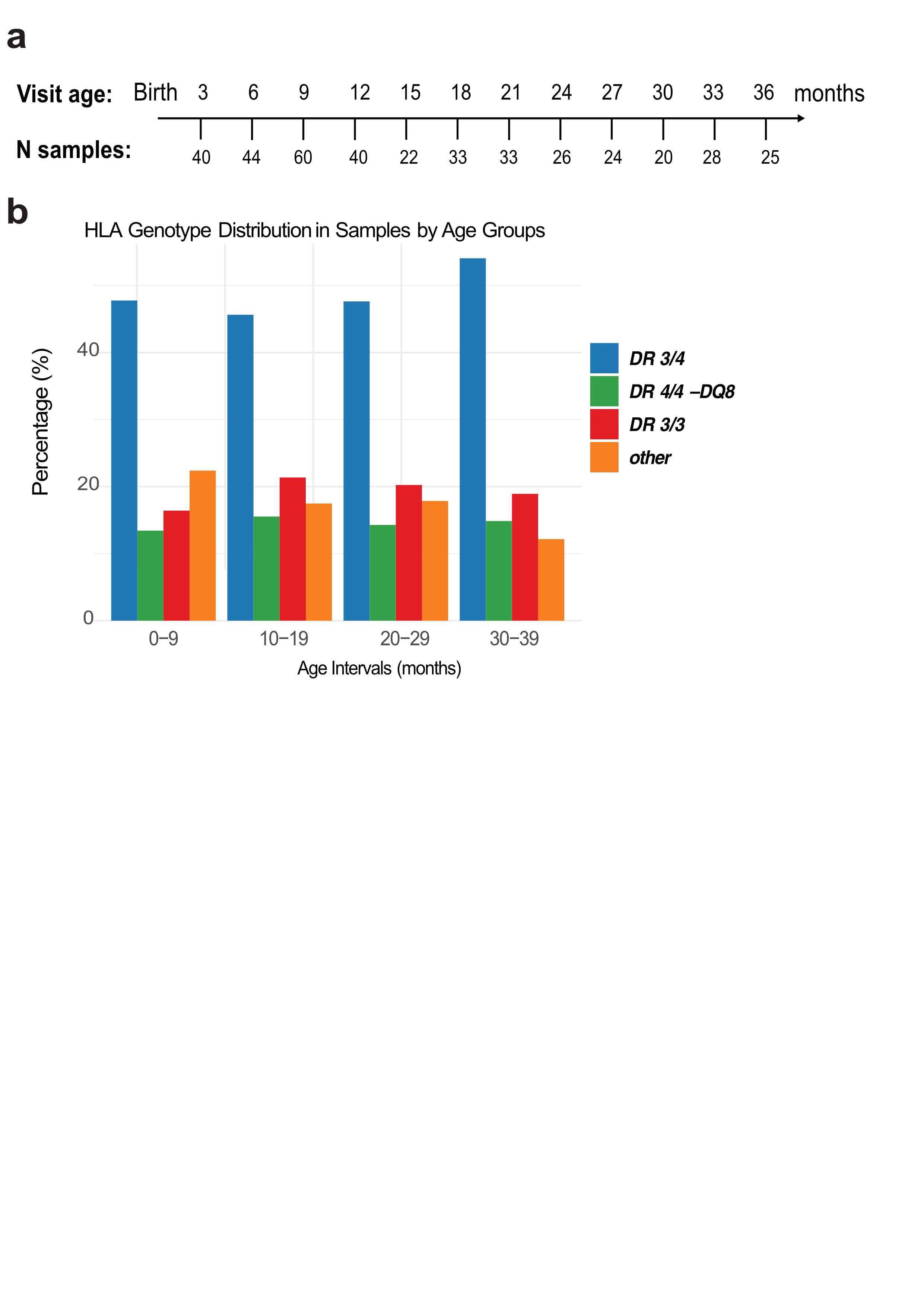


**Supplementary Table 1: Subject Characteristics**

| **Characteristics** | |
| --- | --- |
| **Sex, N (%)** | |
| Female | 64 (59%) |
| Male | 44 (41%) |
| **HLA Genotype, N (%)** | |
| *DR3/DR4-DQ8* | 47 (43%) |
| *DR4-DQ8/DR4-DQ8* | 19 (17%) |
| *DR3/DR3* | 21 (19%) |
| Other | 21 (19%) |
| **Number of Samples per Child,  Median (Interquartile Range)** | 3 (2-5) |

**Supplementary Table 2: See separate Excel file**

**Supplementary Table 3: See separate Excel file**

**Supplementary Table 4: Enrichment of T1D Susceptibility and MHC Genes in Differentially Expressed Gene (DEG) Clusters**

|  | **T1D Susceptibility Genes (n=116; 0.6%**^a^**)** | | | **MHC region Genes (n=159; 0.8%**^a^**)** | | |
| --- | --- | --- | --- | --- | --- | --- |
| **Group** | Number of DEGs (%)^b^ | OR (95% CI) | *P* | Number of DEGs (%)^b^ | OR (95% CI) | *P* |
| Whole DEG Set (n=2432) | 26 (1.1%) | 2.0 (1.3 - 3.2) | 0.002 | 31 (1.3%) | 1.7 (1.1 - 2.5) | 0.01 |
| Cluster A (n=809) | 6 (0.7%) | 1.3 (0.5 - 2.8) | 0.35 | 10 (1.2%) | 1.6 (0.7 - 3.0) | 0.16 |
| Cluster B (n=432) | 9 (2.1%) | 3.8 (1.7 - 7.5) | 0.001 | 13/432 (3%) | 4.0 (2.1 - 7.1) | <0.0001 |
| Cluster C (n=807) | 7 (0.9%) | 1.5 (0.6 - 3.2) | 0.2 | 7/807 (0.9%) | 1.1 (0.4 - 2.3) | 0.84 |
| Cluster D (n=187) | 1 (0.5 %) | 0.9 (0.0 - 5.1) | 0.68 | 0/187 (0%) | 0.0 (0.0 - 2.4) | 0.41 |
| Cluster E (n=68) | 0 (0 %) | 0.0 (0.0 – 9.4) | 1 | 1/68 (1.4%) | 1.8 (0.1 - 10.6) | 0.43 |
| Cluster F (n=129) | 3 (2.5%) | 4.0 (0.8 - 12.4) | 0.04 | 0/129 (0%) | 0.0 (0.0 - 3.5) | 0.64 |

^a^The frequency of the T1D susceptibility genes and MHC region total genes out of the total genes (19,424) represented in the dataset

^b^The frequency of the T1D susceptibility DEG genes and MHC region total DEG genes out of the total DEG genes or total DEG genes in Cluster


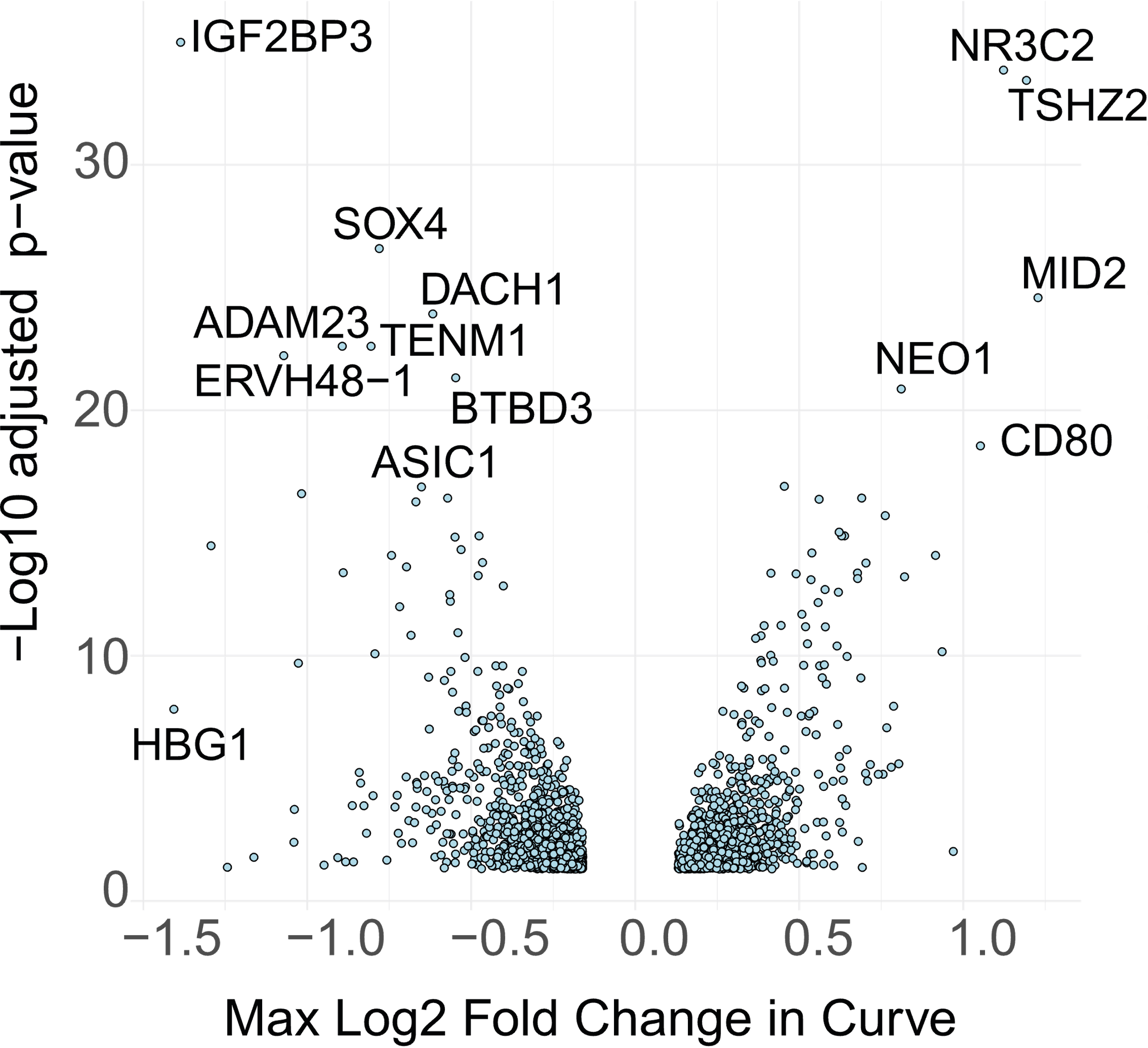


**Supplementary Figure 2:** Volcano plot displaying the maximal log2 fold change in gene expression curves over age and the -log10 adjusted p-value from linear regression modelling of gene expression patterns.

**Supplementary Figure 3:** Heatplot of 15 enriched Gene Ontology Biological Process terms in subgroup Cluster 6 within Cluster B and their associated genes**.**


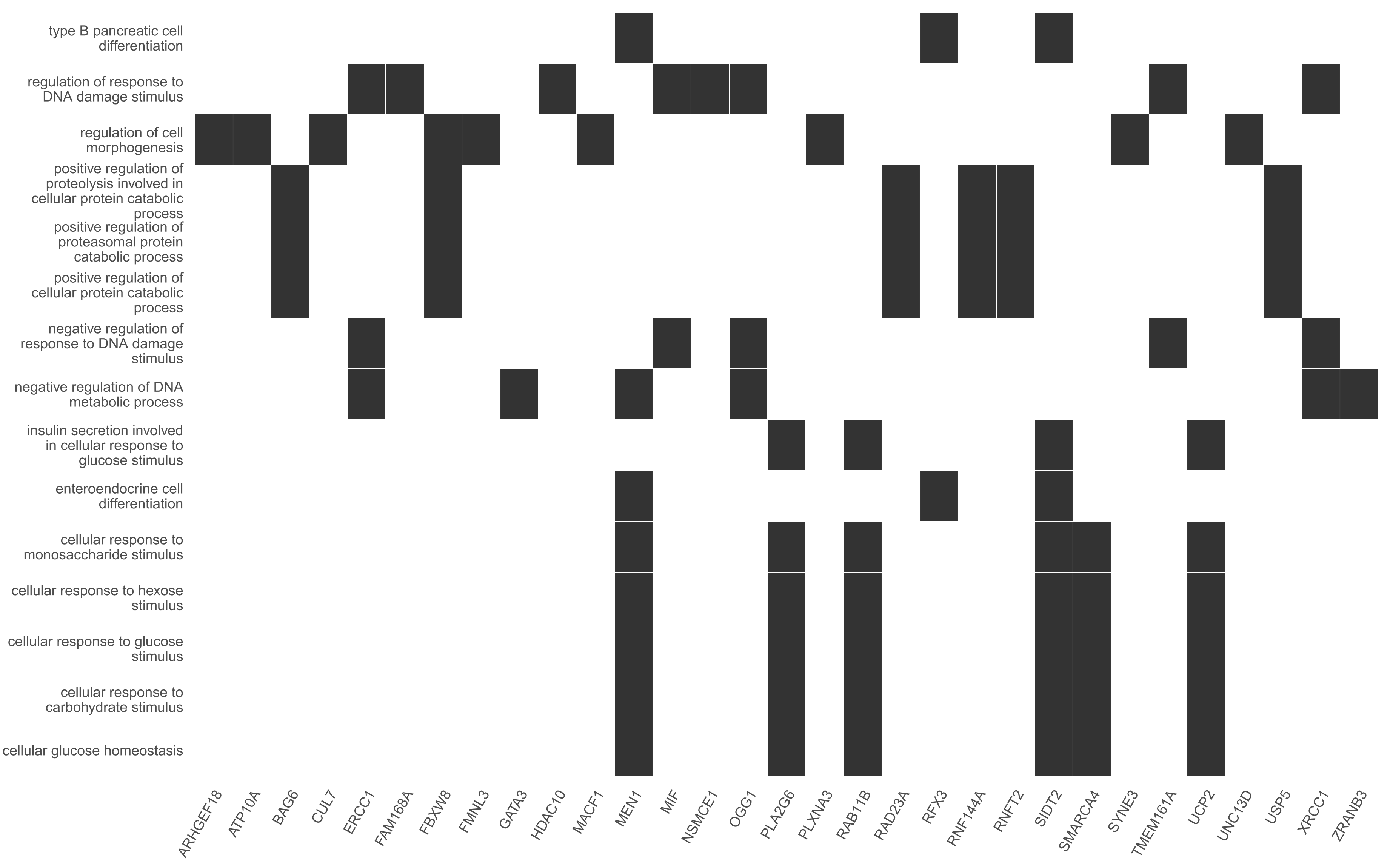


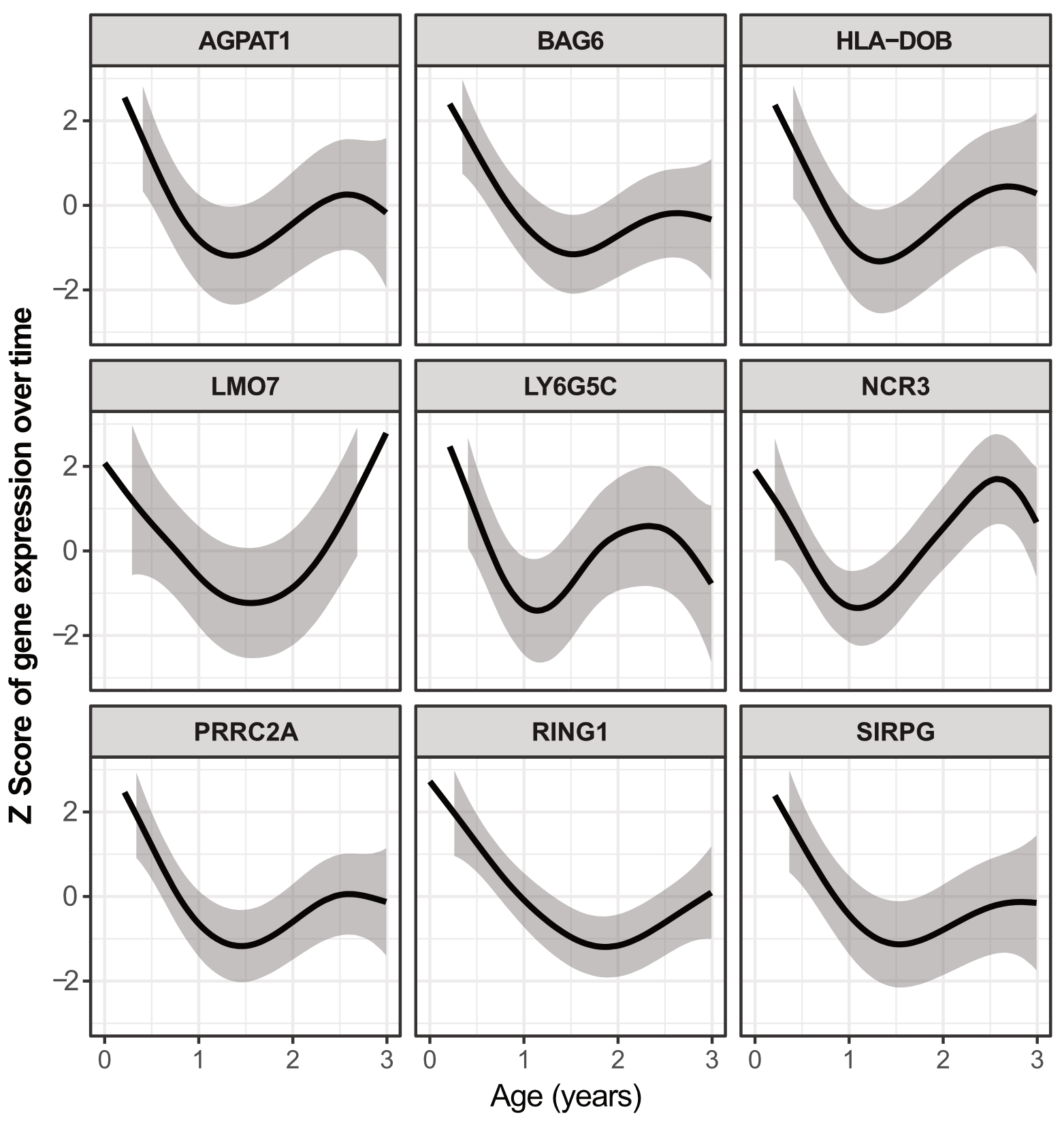
**Supplementary Figure 4***:* **Gene Expression Trajectories for T1D Susceptibility Genes in Cluster B**. This figure illustrates the Z-score trajectories of gene expression over time (in years of age) for the nine T1D susceptibility genes identified in Cluster B, with shaded regions representing the 95% confidence intervals.
